# Self-Locking Polymeric Clips Are Safe for the Closure of Appendiceal Stump in Laparoscopic Appendectomy

**DOI:** 10.3390/medicina59030533

**Published:** 2023-03-09

**Authors:** Kristina Marcinkeviciute, Raminta Luksaite-Lukste, Eugenijus Jasiunas, Tomas Poskus

**Affiliations:** 1 Faculty of Medicine, Vilnius University, LT-03101 Vilnius, Lithuania; 2 Department of Radiology, Nuclear Medicine and Medical Physics, Institute of Biomedical Sciences, Faculty of Medicine, Vilnius University, LT-03101 Vilnius, Lithuania; 3 MMath, Vilnius University Hospital Santara Clinics, LT-08661 Vilnius, Lithuania; 4 Center of Abdominal Surgery, Clinic of Gastroenterology, Nephrourology, and Surgery, Institute of Clinical Medicine, Faculty of Medicine, Vilnius University, LT-08661 Vilnius, Lithuania

**Keywords:** appendicitis, appendectomy, stump closure, polymeric clips, endoloops

## Abstract

***Background*:** Closure of the appendix stump is critical to avoid serious postoperative complications. There are a number of options, but the best one has not been identified yet. The purpose of this study is to evaluate the outcomes of appendiceal stump closure using self-locking polymeric clips and endoloops. ***Methods*:** A retrospective analysis of the prospectively maintained database of patients with acute appendicitis was performed. Patient demographic details and surgical characteristics, including the duration of hospital stay, postoperative complications, and also the cost of the appendix stump closure, were recorded. Patients were divided into two groups according to the appendix stump closure method: the clips group if it was closed with self-locking polymeric clips and the loops group if Vicryl or PDS loops were used. Statistical analysis was performed using Pearson’s chi-squared test, Wilcoxon rank sum (Mann–Whitney U) test, and Fisher’s exact test in R statistical software package version 4.2.1. ***Results*:** 515 patients were included in the study from June 2016 to April 2021. There were no significant differences in terms of demographics (*p*-value in comparison of groups’ sex > 0.99, age *p*-value 0.16), postoperative complications (*p*-value > 0.99), histological findings (*p*-value 0.27), or length of hospital stays (*p*-value 0.18) between the two patient groups (clips group, N = 454 and loops group, N = 61). The price of operation while using different appendiceal stump closures is significantly different. In a laparoscopic appendectomy, one stump closure with self-locking clips costs 7.69 €, with Vicryl loops—91.35 €, with PDS loops—96.51 €, and with a stapler—514.50 €. ***Conclusions*:** Self-locking polymeric clips can be used for the safe and effective closure of an appendiceal stump. There were no significant differences in the postoperative time (30 days) or complication rates among patients in both (clips and loops) groups. Thus, this might be a technique to reduce expenses while maintaining good postoperative results after laparoscopic appendectomy.

## 1. Introduction

Appendicitis is a common surgical cause of acute abdomen and affects approximately 7% of the population in their lifetime [1]. Laparoscopic appendectomy is a gold standard for acute appendicitis [2]. Laparoscopic appendectomy is superior to open appendectomy due to shorter hospitalization time, reduced postoperative analgesic requirement, early food tolerance, quicker return to work, and lower risk of wound infection [3,4]. Moreover, laparoscopy allows for greater visualization and identification of various abdominal disorders that might simulate acute appendicitis, especially if the vermiform appendix is found nonaltered [5,6,7,8]. Closing the appendiceal stump is a critical step in an appendectomy to avoid major complications, such as postoperative fistula, peritonitis, and sepsis [9]. Several closure techniques are reported in the literature, including using a stapler, endoloop, titanium clips, nonabsorbable polymer clips (hem-o-Lok clip), handcrafted loops, transsection using Ligasure, bipolar cautery, or harmonic scalpel [10,11,12]. In recent years clipless/sutureless techniques using a harmonic scalpel for appendiceal stump closure gained popularity and were also proven to be safe and sufficient [12]. A recent systematic review concluded that in terms of the length of stay and postoperative complications, such as wound infection or postoperative ileus, a harmonic scalpel is similar. It has the only advantage of reducing the duration of the operation in comparison with conventional laparoscopic appendectomy techniques [13]. The main drawback of the harmonic scalpel is the price of disposable hand equipment [13]. However, there is no consensus on the technical method for closing the appendix stump. Moreover, the main disadvantage of laparoscopic appendectomy is the higher cost of the surgery due to the expensive equipment used compared to open surgery [14]. Despite great postoperative results, due to the high cost, there is no clinical evidence to justify the regular use of endoscopic staplers [15]. The routine practice of our department is to perform the closure of the appendiceal stump using polymeric self-locking clips. A polymeric clip is a V-shaped clip made of a nonabsorbable polymer that is available in a variety of sizes. It is utilized to seal off bleeding arteries or tissue structures. In laparoscopic surgeries, it may ligate tissue up to 10 mm using a 5 mm trocar or up to 16 mm through a 10 mm trocar [16]. We use three polymeric clips in laparoscopic appendectomy in our center: Two clips are placed on the base of the appendicitis while a third clip is above the appendicitis, and finally, we cut between the clips and remove the appendicitis. In cases when polymeric clips cannot be safely applied on the appendiceal stump (when the diameter of the vermiform appendix is too large for polymeric clips, or it is technically difficult to apply them), endoloops are used. Vicryl (polyglactin) or PDS (polydioxanone) can be used to make a loop ligature. When the loop is in place, the loop is tightened, thereby pinching and securing the knot [16]. We use three PDS loops as a standard or a mix of one PDS and two Vicryl loops due to it being more cost-effective. Since the use of polymeric clips is not a standard treatment method for all appendiceal stump closure in laparoscopic appendectomy [17], our study aimed to evaluate its effectiveness and safety for appendiceal stump closure.

## 2. Methods

### 2.1. Study Population

We performed a retrospective analysis of the prospectively maintained database. The study was approved by the Vilnius Regional Ethics Committee (2019/3-1107-610) in March 2019. All patients treated for acute appendicitis at a tertiary care hospital of Vilnius University Santara clinics (Lithuania) were entered into the prospectively maintained database [18]. Patients who underwent laparoscopic surgery for acute appendicitis from June 2016 to April 2021 and met the inclusion criteria (Table 1) were included in this study. The diagnosis of acute appendicitis was made according to the basis of clinical examination, the leukocytosis and high C-reactive protein in laboratory tests, transabdominal ultrasound, and computed tomography if the diagnosis was suspected clinically, but there were not enough signs to confirm or exclude the diagnosis of acute appendicitis. In this study, patients were not divided into groups according to the appendiceal stump closure technique before the operation. As it is a retrospective analysis of patients who were prospectively registered in the database, our study had no impact on the assignment of patients to one group or another. When laparoscopic appendectomies were introduced in Lithuania, polymeric clips were used to close the appendiceal stump, and they are still applied as a standard in our hospital today. Therefore, alternatives for polymeric clips (endoloops or stapler) for appendiceal stump closure are most commonly used when the surgeon decides at the time of surgery that the closure of the appendiceal stump is not possible with a polymeric clip. As a standard in our center, endoloops are used due to their lower price compared with staplers.

### 2.2. Data Collection

Information obtained from the database included patient demographic characteristics, such as sex and age, with surgical characteristics, including closure method of the appendiceal stump (self-locking polymeric clips or endoloops), with outcomes, such as complications, histological findings, and length of stay, as well as financial outcomes of stump closure method. The patients were divided into two groups based on the performed stump closure technique: self-locking polymeric clips (clips group) and endoloops (loops group). A 30-day follow-up was completed for all patients.

### 2.3. Hypothesis and Outcomes Measures

We hypothesized that self-locking clips and endoloops are equally safe and effective for appendiceal stump closure. The primary outcome of this study was the rate of complications in the two groups: the clips group the and endoloops group. The secondary outcomes were sex, age, histological findings, hospitalization length, Clavien–Dindo grade, and the cost of operation.

### 2.4. Operative Technique

After the induction of general anesthesia, patients were placed supine position. A Veress needle was introduced below the umbilicus, and CO_2_ was insufflated at a pressure of 10–12 mm Hg. In all cases, laparoscopic appendectomy was performed with 3 inserted trocars (5 mm, 10 mm, and 12 mm trocars). The appendicitis was identified, periappendiceal or pericecal adhesions were lysed, and the mesoappendix was dissected with the polymeric or titanium clip placed on the appendiceal artery. The base of the appendicitis was ligated with 3 XL polymeric clips or with 3 PDS (or in some cases, 3 Vicryl loops or 1 PDS and 2 Vicryl loops) according to the surgeon’s choice. Then, appendicitis was divided with the scissors distal to the clips or loops and removed through a 12 mm port.

### 2.5. Statistical Analysis

Statistical analyses were performed using R statistical software package version 4.2.1 (23 June 2022 © The R Foundation for Statistical Computing), PBC, Rstudio 1 July 2022 Build 554 © 2009–2022 Rsudio, PBC. Interval and ratio variables were described by means and standard deviation (SD), by medians, first quartiles (Q1), and third quartiles (Q3). Nominal and ordinal variables were characterized by frequencies and percentages across the corresponding subset of the sample. Shapiro–Wilk and Kolmogorov–Smirnov (K–S) tests we used to check the data for normality.

To compare differences between two independent groups when the variables in these groups were either ordinal or continuous but not normally distributed, we used Pearson’s chi-squared test; Wilcoxon rank sum (Mann–Whitney U) test; and Fisher’s exact test.

To measure the effect size and direction between a dichotomous (binary) variable and a continuous variable, unsatisfying the condition for normal distribution, we used the rank biserial correlation (rrb). To measure the association of categorical variables, we used Cramér’s V effect size. We will assume that when rrb (Cramér’s V) = 0.01–<0.30, we have a small effect; when rrb (Cramér’s V) ≥ 0.31–<0.60, we have a moderate effect; and when rrb (Cramér’s V) ≥ 0.61–1.00, we have a large effect.

## 3. Results

**Figure 1:**
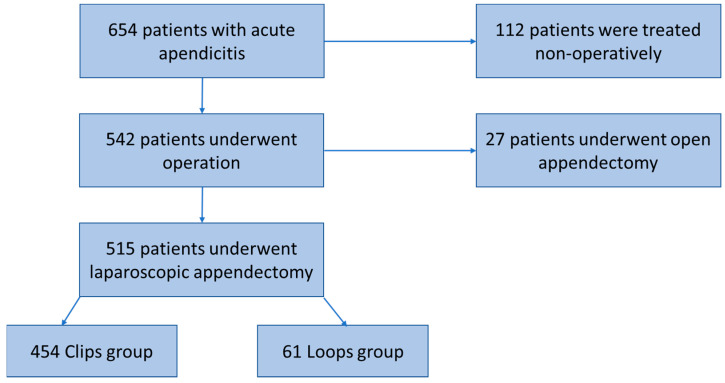
Flowchart of patients’ selection.

Six hundred and fifty-four patients with acute appendicitis diagnosis were first identified. A total of 542 patients underwent an operation, while 112 were treated conservatively and were not included in this study. A total of 27 patients were excluded because they underwent open appendectomy due to conversion after the complicated laparoscopic operation, and 515 patients were finally included in the study (Figure 1).

They were divided into two groups according to the choice of closure methods of the appendiceal stump in the performed laparoscopic appendectomy: self-locking polymeric clips (clips group) (454 patients) and endoloops (loops group) (61 patients).

The demographic and clinical characteristics of the groups are presented in Table 2. There were no statistically significant differences between the groups in sex, age, or histological findings.

A total of 515 patients underwent laparoscopic appendectomy and were followed 30 days after the operation. Table 3 presents the outcomes of the groups. There were no statistically significant differences in outcomes between the clips group and the loops group (*p*-value > 0.99). The 30-day complication rates were low at 1.8% and 1.6% in each group, respectively.

Table 4 presents the exact complications, Clavien–Dindo (C–D) grade, and histological diagnosis of each complication in both (clips and loops) groups. Hemoperitoneum was treated with a repeated operation. The blood was removed from the abdominal cavity; however, a clear source of bleeding was not identified. Abscesses were drained percutaneously. The abdominal wall ligature fistula was treated conservatively and healed. Wound infection, typhlitis, or postoperative fever was treated conservatively with a 10-day course of antibiotics (amoxicillin with clavulanic acid with metronidazole).

The costs of the appendiceal stump closures are presented in Table 5. We calculated the hypothetical costs of the procedures. We use 3 polymeric clips in one laparoscopic appendectomy, while 5 clips in one unit cost 7.69 €, and we use 3 Vicryl or PDS loops, which are packaged one at a time, and one loop costs 30.45 € and 32.17 €, respectively. If all appendiceal stump closures were performed using clips, the cost of stump closure of one operation performed would be 7.69 €; if Vicryl loops were used, the cost would be 91.35 €; if PDS loops were used, the cost would be 96.51 €, as 3 loops are used; and if a stapler was used, the cost would be 514.50 €.

## 4. Discussion

We found that the appendiceal stump can be safely closed using self-locking polymeric clips. The rate of postoperative complications in patients using clip closures and loop closures was low and neither clinically nor statistically significantly different (1.8 vs. 1.6%). Moreover, there are no statistically significant differences or any more significant effect sizes between the two groups of different closure methods of the appendix stump and histological findings, patients’ sex, patients’ age, and the number of hospitalization days.

Acute appendicitis is still often misdiagnosed, and the negative appendectomy rate remains high [19]. In most studies, the negative appendectomy rate is less than 10% [20,21], but in some reported studies, it is higher—about 10–15% [9] and can exceed 20% [19]. A negative appendectomy rate is not reported in our study because in our database [18], only patients with a confirmed diagnosis of acute appendicitis who underwent laparoscopic appendectomy were included. Patients with a negative diagnosis of acute appendicitis were not included in the database; therefore, we could not count the rate of negative appendectomy. However, our hospital’s prior experience revealed a 22.9% negative appendectomy rate when the diagnosis was based on laboratory tests and abdominal ultrasound [19]. The World Society of Emergency Surgery (WSES) recommends that an abdomen ultrasound should be used as the first-line radiological modality for suspected acute appendicitis [22]. However, in recent years, a decreasing trend of negative appendectomies has been observed due to using improved diagnostics tools, such as CT scans and Alvarado scoring, additionally as laboratory tests and abdomen ultrasounds [18,23,24,25]. Therefore, since January 2016, a CT scan was added to the diagnostic protocol in patients in whom diagnosis cannot be made using ultrasound. Since then, we reported a negative appendectomy rate of 4.2% [18]. If the correct diagnosis is made, unnecessary investigations and surgery are avoided. Thus, both additional hospital costs and potential complications during operation are avoided.

In the recent decade, nonoperative management of acute appendicitis is gaining more popularity due to reducing the possible number of complications after laparoscopic appendectomy and especially since some of them can be false positives and unnecessary [26]. Only those patients with a clinical diagnosis of localized appendicitis who do not have diffuse peritonitis or radiological signs of a large abscess, phlegmon, perforation, or tumor could be managed nonoperatively with an antibiotics course [27]. However, nonsurgical treatment of nonperforated appendicitis is not standardized and is used only in trials [28]. Moreover, nonoperative treatment was related to a higher rate of abscess, readmission, and total healthcare costs in comparison to surgical treatment and the cost of operation and possible complications [29]. Therefore, surgical treatment of acute appendicitis still remains the first-line choice for the management of acute appendicitis.

Appendiceal stump closure is a critical step in appendectomy operation, as inappropriate appendiceal stump closure might result in major postoperative complications. A variety of stump closure methods in laparoscopic appendectomy are used in clinical practice. The most popular are loops, stapling devices, clips, or electrothermal devices [30]. However, there is no agreement on the technical method that should be used when closing the stump. Based on a recommendation for the choice of appendix stump closure, only the stapler should be used if the appendix base is inflamed. If the entire appendix cannot be visualized, a clip or stapler should be chosen. In addition, only if the appendix is mobilized, a clip, loop, or stapler can be used depending on the base thickness [17]. A recently performed meta-analysis showed that endoloops and endostaplers are safe for appendix stump closure and have no difference in postoperative complication rate [31]. While self-locking polymeric clips (also known as Hem-o-Lok clips) are a less prevalent option in laparoscopic appendectomy for stump closure and are more used for the closure of bleeding vessels or tissue structures [16], they were also proven to have advantages: They are safe, cheap, and easy to use [32]. Although the clip in the literature is considered to be an unsafe choice for an inflamed appendix base [17], or there is a need for further high-quality studies before polymeric clips can be suggested as the gold standard for appendiceal stump closure [33], 131 (29.3%) patients in the clips group in our study were with gangrenous or perforated appendicitis where appendix base is usually inflamed, and only 4 of them had postoperative complications. According to clinical trial findings, all technical variants, endoloop, self-locking polymeric clips, and endostapler, are equivalent in terms of postoperative complications [34,35]. The presented deep surgical site complication rate was 1.7% of patients [34]. Our study presents comparable complication rates—1.8% for self-locking polymeric clips and 1.6% for endoloops. Moreover, the shortest operative time and the lowest price of the operation were noted in the study [34] if self-locking polymeric clips were used. Comparable results report recent studies where polymeric clips are cheaper and less time-consuming alternatives to other commonly used techniques, such as endoloops [16,36,37,38,39,40,41]. In one study [37], a lower rate of intra-abdominal surgical abscesses was reported while using polymeric clips. Another advantage of polymeric clips is the ability to apply them, as most surgeons have several years of experience with laparoscopic cholecystectomies where these polymeric clips are used [35].

Patients undergoing laparoscopic appendectomy had many advantages of short operative time, the possibility to examine other abdominal organs, shorter hospitalization, and lower rates of postoperative complications compared to open appendectomy [42,43,44,45]. Recent studies show that the conversion rate from laparoscopic appendectomy to open appendectomy is 2–4% [46,47]. Conversion rates may vary between studies due to differences in the frequency of previous surgeries and adhesions, ages, and varying severity of appendicitis [46]. According to our study, the rate is 5% (27 patients) (Figure 1). These patients were not included in our study, as we were focused on two techniques’ (polymeric clips and endoloops) comparison of safety in laparoscopic appendectomies.

However, the main drawback of laparoscopic appendectomy discussed in the literature is the high price of it due to expensive equipment [15]. We compared the prices in our hospital and found that self-locking polymeric clips are 11.9 times cheaper than Vicryl, 12.55 times more than PDS loops, and 66.9 times cheaper than Echelon staplers per one laparoscopic appendectomy operation. Moreover, from 61 cases where endoloops were used in our center, Vicryl loops were used 18 times (30%), and the mixed-loop technique (Vicryl and PDS loops) was chosen 8 times (13%) due to the lower price compared to only using the standard PDS loops. Therefore, polymeric clips are not only safe and effective for perforated and nonperforated appendicitis, but they are also cost-effective. Moreover, a recent study reports that polymeric clip choice as an alternative to endoloops or staplers does not prolong operation and hospitalization time [48]. It could be an option to reduce the price of laparoscopic appendectomy.

Surgery is one of the most energy-intensive activities, actively contributing to climate change [49]. Reprocessing and remanufacturing allow for the reuse of some of the medical devices. Self-locking polymeric clips are used, and the small package could be recycled. However, staplers are large polymeric/metal instruments, which in most instances, would end in landfills and would not be reused or recycled. The package of Vicryl or PDS loops are similar to clips in terms of recycling and much smaller than staplers [50,51].

### Limitations

This is a retrospective analysis of a prospectively maintained database, so all drawbacks of a retrospective study are applied here. We did not count operative time. As laparoscopic appendectomy is one of the first operations performed by residents, we feel that the different time is related more to surgical experience than to the technique of appendiceal stump closure. From our database, it is not possible to distinguish whether the surgery was performed completely by the resident or by the attending physician. Another limitation of this study is the heterogeneous patient groups. Our study had no effect on the assignment of patients to one of two groups. In our hospital, polymeric clips for appendiceal stump closure are standard practice, and loops were used only in cases where polymeric clips were judged to be difficult to apply. However, we believe that even having these limitations, it is still possible to safely recommend further use of polymeric clips for the closure of the appendiceal stump.

## 5. Conclusions

Self-locking polymeric clips for appendiceal stump closure are as safe as endoloops. As there were no significant differences in the postoperative period and complication rates among these patients, this might be a way to reduce hospital costs while maintaining good postoperative outcomes after laparoscopic appendectomy. Our retrospective analysis with a representative number of patients highlights the feasibility of polymeric clips for the closure of the appendicitis stump in laparoscopic appendectomy. Our study also highlights the huge cost difference, ranging from 11.9 to 66.9 times lower cost, when using polymer clips instead of their alternative—loops (PDS or Vicryl) or stapler.

## Figures and Tables

**Table 1:** Inclusion and exclusion criteria for a retrospective study.

Inclusion Criteria	Exclusion Criteria
Adults ≥ 18 years of age	Patients treated conservatively
Patients with acute appendicitis diagnosis	Patients underwent open appendectomy
Patients underwent laparoscopic appendectomy	Patients who needed a stapler application

**Table 2:** Clinical characteristics of two groups (clips group and loops group) of patients who underwent laparoscopic appendectomy.

			Closure Method of the Appendix Stump		
Variable	N	Overall, N = 515 ^1^	Clip, N = 454 ^1^	Loop, N = 61 ^1^	*p*-Value ^2^
Sex	515				>0.99
Female		245 (48%)	216 (48%)	29 (48%)	
Male		270 (52%)	238 (52%)	32 (52%)	
Age	515	37 (15)	36 (15)	40 (17)	0.16
Histological findings	515				0.27
Early changes		15 (2.9%)	14 (3.1%)	1 (1.6%)	
Secondary changes		6 (1.2%)	5 (1.1%)	1 (1.6%)	
Phlegmonous appendicitis		338 (66%)	304 (67%)	34 (56%)	
Gangrenous appendicitis		105 (20%)	89 (20%)	16 (26%)	
Perforated appendicitis		51 (9.9%)	42 (9.3%)	9 (15%)	

^1^ Mean (SD) or Frequency (%). ^2^ Pearson’s chi-squared test; Wilcoxon rank sum test; and Fisher’s exact test.

**Table 3:** Characteristics of patients’ groups and complications characteristics.

			Closure Method of the Appendix Stump		
Variable	N	Overall, N = 515 ^1^	Clip, N = 454 ^1^	Loop, N = 61 ^1^	*p*-Value ^2^
Complications	515				>0.99
Complications: No		506 (98%)	446 (98%)	60 (98%)	
Complications: Yes		9 (1.7%)	8 (1.8%)	1 (1.6%)	
Length of stay	515	3.15 (1.81)	3.09 (1.72)	3.61 (2.36)	0.18

^1^ Mean (SD) or Frequency (%). ^2^ Pearson’s Chi-squared test; Wilcoxon rank sum test; Fisher’s exact test.

**Table 4:** Complications of the patients in the groups. C–D grade—complication grade according to Clavien–Dindo classification.

Clips Group, N = 454			Loops Group, N = 61		
Complication	C–D Grade	Histological Diagnosis	Complication	C–D Grade	Histological Diagnosis
Wound infection	1	Phlegmonous appendicitis	Postoperative fever	1	Perforated appendicitis
Hemoperitoneum, anemia	3b	Early changes			
Intra-abdominal abscess	2	Secondary changes			
Intra-abdominal abscess	2	Perforated appendicitis			
Abdominal wall ligature fistulae	1	Phlegmonous appendicitis			
Intra-abdominal abscess	2	Gangrenous appendicitis			
Typhlitis	2	Perforated appendicitis			
Postoperative fever	1	Perforated appendicitis			

**Table 5:** Cost of different closure methods of appendix stump.

Closure Methods of the Appendix Stump	Cost (€)/Unit	Cost (€)/Surgery	Cost (€)/515 Patients
Self-locking polymeric clip	7.69	7.69	3960.35
Vicryl loop	30.45	91.35	47,045.25
PDS loop	32.17	96.51	49,702.65
Echelon stapler (FlexTM 45)	514.50	514.50	264,967.50

## Data Availability

The data presented in this study are openly available. https://doi.org/10.3390/jcm10112456 (accessed on 8 December 2022), reference number in this article—[16].
